# Late gadolinium enhancement is compatible with advanced age in hypertrophic cardiomyopathy: implications for risk stratification of sudden death

**DOI:** 10.1186/1532-429X-14-S1-P156

**Published:** 2012-02-01

**Authors:** Raymond H Chan, Martin Maron, Susie Hong, Tammy S Haas, John Lesser, C Michael Gibson, Warren J Manning, Barry J Maron, Evan Appelbaum

**Affiliations:** 1 BIDMC, Boston, MA, USA; 2 Tufts Medical Center, Boston, MA, USA; 3 Minnesota Heart Institute Foundation, Minneapolis, MN, USA

## Background

Hypertrophic cardiomyopathy (HCM) is most common in patients under 40 years and is associated with early sudden death, but HCM is increasingly recognized in elderly patients. We sought to characterize and identify features unique to elderly HCM patients when compared with youthful patients using CMR.

## Methods

Cine CMR and late gadolinium enhancement (LGE) imaging were performed in 902 HCM patients, including 202 young (15-35 years; mean 24.5 ± 6.3 years), and 143 elderly patients (age > 65 years; mean 70.9 ± 4.8 years).

## Results

Elderly HCM patients had a lower proportion of males (53.8% elderly vs 70.3%, p=0.002). They had significantly lower left ventricular (LV) mass (153 ± 64g elderly vs 183 ± 90g, p<0.001) and lower maximal LV wall thickness (19 ± 4mm elderly vs 21±6mm, p<0.001). The most common maximal thickness segment in the elderly patients was in the basal anterior septum (35% elderly vs 22% , p=0.01). Elderly patients were also more likely to have the thickest segment located within the septum (OR 1.74, 95% C.I. 1.12 - 2.69, p=0.01). Elderly patients were far less likely to have massive hypertrophy (maximal wall thickness >30mm) (OR 0.15, 95% C.I. 0.03-0.64, p=0.01).

Elderly HCM patients had a smaller LV cavity size, with lower LV end-diastolic volume (138 ± 41ml elderly vs 165 ± 45ml, p<0.001) and smaller LV end-diastolic dimension (52 ± 7mm elderly vs 54 ± 7mm, p=0.05). The elderly patients had similar LV ejection fraction (LVEF) (67± 11% elderly vs 66± 9%, p>0.1) and similar rates of depressed (<60%) LVEF ( 21% elderly vs 20%, p=0.79).

The prevalence of LGE (a marker of fibrosis) was similar in both groups (40.9% elderly vs 43.5%, p=0.64). When present, both LGE mass (8.0 ± 14.7g elderly vs 9.6 ± 15.1g, p>0.10) and percentage of LV with LGE (8.6 ± 10.4% elderly vs 6.9 ± 8.3%, p>0.10) were similar.

## Conclusions

Substantial amounts of late gadolinium enhancement are compatible with normal longevity in many patients with hypertrophic cardiomyopathy. Presence of LGE may thus have different prognostic significance across the vast spectrum of HCM. This has potential implications for using LGE as a risk stratification tool for sudden death in HCM.

## Funding

Nil.

